# Negative Affect Mediates Effects of Psychological Stress on Disordered Eating in Young Chinese Women

**DOI:** 10.1371/journal.pone.0046878

**Published:** 2012-10-05

**Authors:** Jue Chen, Zhen Wang, Boliang Guo, Jon Arcelus, Haiyin Zhang, Xiuzhen Jia, Yong Xu, Jianyin Qiu, Zeping Xiao, Min Yang

**Affiliations:** 1 Shanghai Mental Health Center, Shanghai Jiao Tong University School of Medicine, Shanghai, China; 2 Institute of Mental Health, University of Nottingham, Nottingham, United Kingdom; 3 Loughborough University Centre for Research into Eating Disorders (LUCRED), Loughborough, Leicestershire, United Kingdom; 4 Leicester Eating Disorders Service, Leicester, Leicestershire, United Kingdom; University of Illinois at Chicago, United States of America

## Abstract

**Background:**

The bi-relationships between psychological stress, negative affect and disordered eating has been well studied in western culture, while tri-relationship among them, i.e. how some of those factors influence these bi-relationships, has rarely been studied. However, there has been little related study in the different Chinese culture. This study was conducted to investigate the bi-relationships and tri-relationship between psychological stress, negative affect, and disordered eating attitudes and behaviors in young Chinese women.

**Methodology:**

A total of 245 young Chinese policewomen employed to carry out health and safety checks at the 2010 Shanghai World Expo were recruited in this study. The Chinese version of the Perceived Stress Scale (PSS-10), Beck Depression Inventory Revised (BDI-II), Beck Anxiety Inventory (BAI), and Eating Attitude Test (EAT-26) were administered to all participants.

**Principal Findings:**

The total scores of PSS-10, BDI-II and BAI were all highly correlated with that of EAT-26. The PSS-10 score significantly correlated with both BDI-II and BAI scores. There was no statistically significant direct effect from perceived stress to disordered eating (–0.012, 95%CI: –.038∼0.006, *p* = 0.357), however, the indirect effects from PSS-10 via affect factors were statistically significant, e.g. the estimated mediation effects from PSS to EAT-26 via depression and anxiety were 0.036 (95%CI: 0.022∼0.044, *p*<0.001) and 0.015 (95%CI: 0.005∼0.023, *p*<0.01), respectively.

**Conclusions:**

Perceived stress and negative affects of depression and anxiety were demonstrated to be strongly associated with disordered eating. Negative affect mediated the relationship between perceived stress and disordered eating. The findings suggest that effective interventions and preventative programmes for disordered eating should pay more attention to depression and anxiety among the young Chinese female population.

## Introduction

From ancient times “food” and “eating” are a vital part of Chinese culture [1] as they have psychological and social function in this society. Food is also used to treat and prevent disease in this culture [2]. However, since China’s ‘open policy’ started two decades ago with increasing access to Western-style media, Chinese society has changed their aesthetic ideals and their relationship with food. Western cultural values and media imagery have been blamed for the increase in body dissatisfaction among this population, which has played a major role in the development of disordered eating and Eating Disorders (ED) in China [3], [4]. This is not unique for China as Westernization of cultures has been linked to disordered eating and ED in other non-Western societies, such as Fiji, Malaysia and Japan [5], [6].

Though there are no current community-based epidemiological data on ED in China, early surveys of Chinese students have demonstrated ED prevalence rates ranging from 1.1 to 3.62% [7], [8]. More recent studies have shown a trend towards an increase of these disorders, ranging from 4.7% to 17% [9], [10]. These values are approaching the prevalence rates of university students in western countries (6% to 20%) [11], [12], [13]. Dieting and binging behaviors, which are most frequent disordered eating behaviors, have been found to be common among Chinese young females. Studies in this area have reported that between 49.9% [14] and 55.7% [8] of the university females had dieted and between 20% [15] and 58.3% [14] of the students binged frequently. Thus, disordered eating has become a significant and growing problem in modern China [3], [15].

Although, the etiology of the eating disorders remains unclear, previous studies have demonstrated that genetic predisposition and specific environmental factors are implicated in the development of these conditions [16], [17]. Among the various factors studied, psychological stress and negative affects, such as depression and anxiety, have been suggested to be strongly associated with disordered eating and ED [18], [19], [20], [21].

Various psychological stressors have been shown to be related to eating problems such as prolonged work stress or stress related to social situation [22], [23]. As resilience to the stress has been shown to be crucial for a sense of well-being, which includes a positive social interactions [24], it is not surprising that the role of stress has been found to determine the onset and the natural course of eating disorders [25]. It is not so much the severity of the stressful event but the perception of the stressful situations which may precipitate a disordered eating. Lazarus proposed that for an event or situation to be considered stressful, it must be perceived as stressful by the individual [26]. Subsequently the Perceived Stress Scale (PSS) [27] have been used widely to measure the way patients perceive stress generally [28], [29]. It measures the degree to which one perceives aspects of one’s life as uncontrollable, and unpredictable. Researchers of western countries have identified an association between the ability of a person to perceived stress and disordered eating [19], [30], [31]; in particular, it has been found that eating-disordered individuals perceive events more stressful than controls [32].

It has also been widely accepted that psychological stress is associated with negative affects such as depression, and anxiety [33], [34], [35]. Furthermore, a strong association has been found between negative affect and disordered eating behaviors [20], [21]. The authors of these studies have demonstrated that negative emotions such as depression, anxiety, anger, or sadness leads to changes in eating behavior, such as a decrease [36] or increase [37] of food intake. Negative affect have been shown to result in disordered eating in not only eating-disordered women [38], but also in normal-weight dieters [39].

Although the bi-relationships between psychological stress, negative affect and disordered eating, i.e. pairwise relationships between psychological stress and disordered eating, between psychological stress and negative affect, and between negative affect and disordered eating, have been well studied in western culture, few study has explored the tri-relationship among them, i.e. how some of those factors influence these bi-relationships.

Obviously, rapid development and change in recent China leads to increasing psychological stress in Chinese population. Compared to Western culture, firstly, Chinese tend to suppress or deny their feelings, which has contributed to the development of psychosomatic symptoms [40], [41]; while disordered eating is a kind of psychosomatic symptom. Secondly, there have been rich eating cultures in the Chinese long history. Accordingly, what is the relationship between stress, feelings and eating in this unique ‘eating culture’? Are Chinese people prone to developing disordered eating behaviors as a coping mechanism when confronted with stress? Nevertheless, there has been few related studies in China.

Based on the results of earlier studies, this study has two aims. The first aim is to examine the relationship between perceived stress and disordered eating in a sample of Chinese young females known to be confronted with high levels of stress, such as policewomen. The second aim is to examine whether the relationship between perceived stress and disordered eating is mediated by symptoms of negative affect, such as depression and anxiety. The study will be conducted in China making it one of the very few studies in the field of eating disorders from this country.

## Methods

### Ethics Statement

This research protocol was approved by the Ethics Committee of the Shanghai Mental Health Center and written informed consent have been obtained from all the study participants.

### Participants

Participants were recruited from policewomen who were employed to carry out the health and safety checks at the 2010 Shanghai World Expo as part of the Chinese army. These women were part of the undergraduate and postgraduate student population who trained for this specific role for 8-hours-per-day for a period of six month before. This study was carried out in July 2010.

The heath care coordinator for the army selected participants randomly for the study by distributing the invitation letter which explained the project in detail. Following agreement to participate in the study, written informed consent was taken from the policewomen. Due to financial pressures the study could only invite a max of 260 participants.

### Instruments and Measures

Participants completed a set of self-administered questionnaires to evaluate their perceived stress, their eating disorders symptomatology and their anxiety and depression. Socio-demographic information was also obtained and participants’ body mass index (BMI) was calculated (kg/m^2^) with their reported height and weight.

#### Perceived stress scale-10 (PSS-10) [42]

The PSS-10 was used to measure the way women perceived stress in their day to day life. It measures the degree to which one perceives aspects of one’s life as uncontrollable, unpredictable, and overloading. Participants are asked to respond to each question on a 5-point Likert scale ranging from 0 (never) to 4 (very often), indicating how often they have felt or thought a certain way within the past month. Scores can range from 0 to 40, with higher composite scores indicative of greater perceived stress. The PSS-10 has demonstrated good reliability and validity in western countries [43], [44]. The psychometric properties of the Chinese version of the PSS-10 also demonstrated adequate psychometric properties for evaluating stress levels, which support its use among the Chinese population [45]. In the present study, the overall Cronbach’s alpha was 0.86.

#### The beck depression inventory revised (BDI-II) [46]

The BDI-II is a 21-item self-administered instrument designed to assess the severity of depression symptoms over the preceding week. Each item is assigned a score of 0–3, with 3 indicating the most severe symptoms. A cumulative score is determined by adding the scores of the individual items. The total score can range from 0 to 63. Higher total scores indicate more severe depression symptoms. The BDI-II is a reliable and well-validated measure in screening for depression symptoms in adults with Cronbach’s alpha ranging from 0.73 to 0.95, and the Simplified Chinese version has been widely used in China [47].

#### The beck anxiety inventory (BAI) [48]

The BAI is a 21-question 4-point self-report inventory that is used for measuring the severity of an individual’s anxiety. Possible scores range between 0 and 63. Increasing scores indicate increasing intensity of anxiety symptoms. The BAI is a widely accepted instrument with high internal consistency (Cronbachs α = 0.92), a test-retest reliability over one week of 0.75, and has been shown to have good validity [49]. The Simplified Chinese version of the BAI has similar psychometric properties [50].

#### 26-Item eating attitude test (EAT-26) [51]

Disordered eating symptoms were assessed using EAT-26. This is one of the most widely used standardized measures used to assess “eating disorder risk” based on attitudes, feelings, and behaviors related to eating and eating disorder symptoms [51], [52]. Participants are asked to respond to each question on a 6-point scale which score respectively 3 (always), 2 (Usually), 1 (Often), 0 (never), 0 (Rarely) and 0 (Sometimes), indicating how often they have the eating symptoms and concerns that are common in eating disorders. Total scores of EAT-26 can range from 0 to 78, with higher scores indicative of more severe disordered eating. A score at or above 20 indicates a high risk of eating disorder. It is widely used and highly reliable and valid, including the Chinese version [4], [51], [52], [53].

### Analytic Strategy

**Figure 1:**
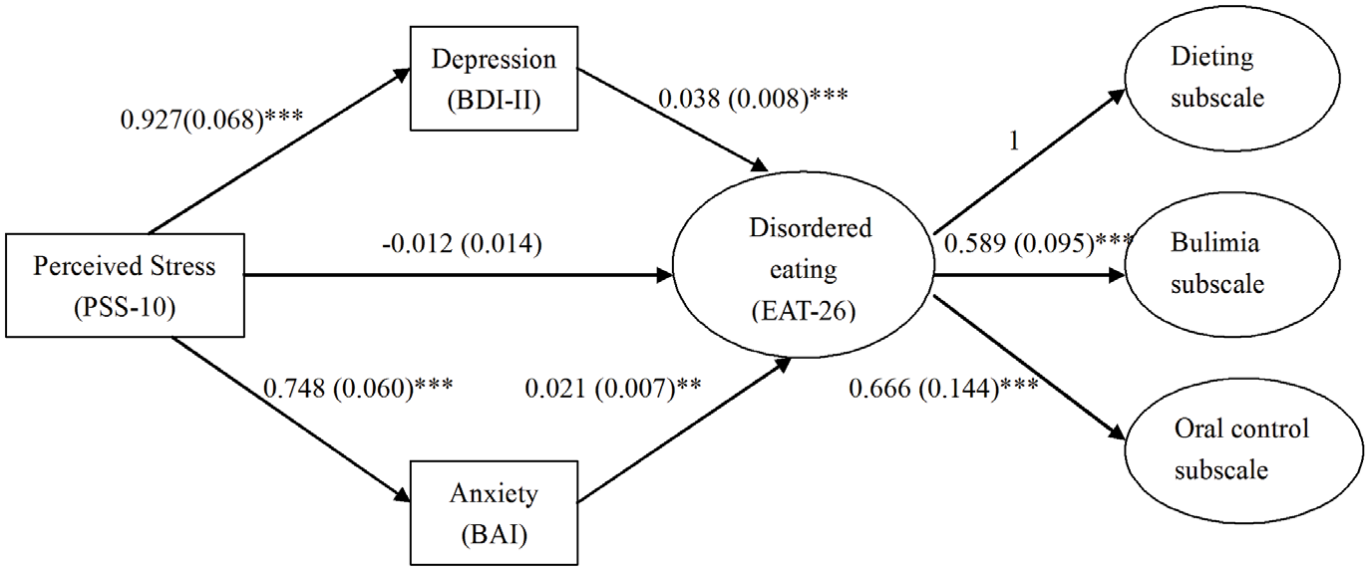
Mediation model exploring the relationship between PSS and EAT. Note1: PSS-10, 10-item Perceived Stress Scale; BDI-II, Beck Depression Inventory Revised; BAI, Beck Anxiety Inventory; EAT-26, 26-item Eating Attitude Test. Note2: *p<0.05; **p<0.01; ***p<0.001. Note3: Indirect effects from PSS to EAT-26 via depression is 0.036 (95%CI: 0.022∼ 0.044, *p*<0.001); Indirect effects from PSS to EAT-26 via anxiety is 0.015 (95%CI: 0.005∼0.023, *p*<0.01).

Descriptive statistics and Pearson’s correlation were used to explore the potential associations among variables included in this study. Path regression analysis was used to test the hypothesized that perceived stress could lead to eating disorder and such relationship could be mediated by affect factors. In the analysis disordered eating was the outcome variable, perceived stress was the predictor and affect factors were mediators as shown in Figure 1. Confirmatory Factor Analysis (CFA) of EAT-26 showed the 2nd order factor structure of disordered eating measure with 1 second order factor and 3 first order factors fitting the data well, therefore latent disordered eating factor was used in the path analysis. Although CFA showed good factor structure for PSS and affect measure, the sample size in this study is not large enough to include all above variables as latent measures in path modeling, so summary measure of item scores of each scale was used for the anlaysis. Mplus software was used to conduct the CFA and path regression analysis [54]. Bootstrapping with 500 draws was used to quantify the 2.5–97.5th percentiles (equivalent to 95%CI) of mediation effects due to its potential non-normality [55].

## Results

### Descriptive Analyses

Out of the 260 recruited participants a total of 245 (94.2%) policewomen completed this study and were brought into statistical analysis. All of the participants were unmarried young policewomen and had the same working hours. The demographic characteristics, i.e. age, educated time and results of the questionnaires are described (Table 1).

**Table 1:** Socio-demographic characteristics and eating disorders, perceived stress values, depression and anxiety scores of the participants studied (n = 245).

Variable	Participants		
	Mean	SD	Range
Age (year-old)	21.14	1.30	18∼25
Educated time(years)	14.47	1.20	11∼18
BMI (kg/m^2^)	21.58	2.14	16.00∼30.82
PSS-10	15.05	5.82	0∼33
BDI-II	8.61	7.98	0∼39
BAI	8.08	7.42	0∼37
EAT-26	10.71	8.76	0∼69

Note: BMI, Body Mass Index; PSS-10, 10-item Perceived Stress Scale; BDI-II, Beck Depression Inventory Revised; BAI, Beck Anxiety Inventory; EAT-26, 26-item Eating Attitude Test.

### Correlations between the Variables

In the total participants the variables of perceived stress, depressive affect and anxious affect were all highly correlated with disordered eating measures (Table 2). In addition, higher level of perceived stress was related with not only higher scores on depression but also higher scores on anxiety.

**Table 2:** Bivariate correlations between the variables for the participants.

Variable	PSS-10	BDI-II	BAI
PSS-10	–		
BDI-II	0.673***	–	
BAI	0.589***	0.707***	–
EAT-26	0.307***	0.408***	0.330***

Note: PSS-10, 10-item Perceived Stress Scale; BDI-II, Beck Depression Inventory Revised; BAI, Beck Anxiety Inventory; EAT-26, 26-item Eating Attitude Test.

* p<0.05;

** p<0.01;

*** p<0.001.

### Mediations Analysis

We predicted that the effect of perceived stress in causing disordered eating would be mediated by negative affect of depression and anxiety. Path regression analysis results showed depression and anxiety could increase the disordered eating measure, and perceived stress strongly influences both depression and anxiety level. There was no statistically significant direct effect from perceived stress to disordered eating (–0.012, 95%CI: –.038∼0.006, *p* = 0.357), however, the indirect effects from PSS via affect factors were statistically significant, e.g. the estimated mediation effects from PSS to EAT-26 via depression and anxiety were 0.036 (95%CI: 0.022∼0.044, *p*<0.001) and 0.015 (95%CI: 0.005∼0.023, *p*<0.01), respectively (Figure 1).

## Discussion

Eating Disorders, once considered a Western “culture-bound” syndrome, is now known to exist among non-Western populations. The last few years have seen a significant increase in disordered eating behaviors in China [3], [8], [15], which is likely to reflect an increase in the incidence rate and awareness of the illness by Chinese professionals.

This study showed that, within the Chinese population of young women, disordered eating is highly associated with perceived stress. This finding is consistent with past studies examining the relationship between stress and eating disorders in the Western world [19], [31], [56]. Results also indicated a strong association between symptoms of depression and anxiety and levels of disordered eating. This again adds to the existing literature from other countries [21], [38]. The findings of this study are also consistent with the psychosomatic theory [57], the affect regulation models [58] and the escape theory [37], which explains disordered eating behaviors. These theories imply that disordered are used to relieve negative emotions and aversive self-awareness [21].

The study also aimed to investigate the mediation effect of negative affect in the form of anxiety and depressive symptoms, in the relationship between perceived stress and disordered eating symptoms. In that respect the study found that, among Chinese women, depression and anxiety mediated this relationship. This could be explained by analyzing the role of certain eating disorders behaviors, for example, it has been suggested that disordered eating behaviors such as binge eating and purging may relieve tension or regulate anxiety and may serve as a form of stress release in young women [37]. These finding are in keeping with Western findings. For example, Rojo and his colleagues (2006) found that psychiatric co-morbidity, including depression and anxiety, was a partial mediating factor in the association between stress and EDs [59]. Another study proposed that the association between certain psychological stress variables, including family conflict, family cohesion and childhood abuse, and disordered eating was mediated by alexithymia and depression [60].

The strong association found between disordered eating and psychological stress, and the mediating role of both of depression and anxiety among this relationship, would increase our understanding of the etiology of disordered eating. It also implies that the existed relationships between stress and disordered eating may weaken when the mediated negative affects of depression and anxiety is controlled. The findings of this study would suggest that interventions aiming at improving depression and anxiety may reduce eating problems among young Chinese women when confronted with stress. In view of the rapid increase of individuals with disordered eating behaviors in China, Chinese professionals and policymakers should aim to develop preventive strategies for the vulnerable population targeting at anxiety and depression symptoms which have been developed as a result of stress factors.

There are several limitations to this study that should be noted. The study is limited by the reliance on self-report measures, the specific population studied, without control groups and the cross-sectional nature of the study which leads to difficulty to determine a causal order among the variables. Further research may address some of the methodological issues raised in this study in order to clarify the complex interrelationships among stress, affect, and disordered eating.
